# A multimodal digital twin for autonomous micro-drilling in scientific exploration

**DOI:** 10.1007/s11548-025-03465-3

**Published:** 2025-06-26

**Authors:** Saul Alexis Heredia Perez, Tze Lun Lok, Enduo Zhao, Kanako Harada

**Affiliations:** 1 Graduate School of Medicine, The University of Tokyo, Hongo 7-3-1, Bunkyo City, 113-8654 Tokyo Japan; 2 Graduate School of Engineering, The University of Tokyo, Hongo 7-3-1, Bunkyo City, 113-8654 Tokyo Japan

**Keywords:** Digital twin, Simulation and animation, Deep learning methods, Robotics and automation in life sciences

## Abstract

**Purpose:**

To support research on autonomous robotic micro-drilling for cranial window creation in mice, a multimodal digital twin (DT) is developed to generate realistic synthetic images and drilling sounds. The realism of the DT is evaluated using data from an eggshell drilling scenario, demonstrating its potential for training AI models with multimodal synthetic data.

**Methods:**

The asynchronous multi-body framework (AMBF) simulator for volumetric drilling with haptic feedback is combined with the Isaac Sim simulator for photorealistic rendering. A deep audio generator (DAG) model is presented and its realism is evaluated on real drilling sounds. A convolutional neural network (CNN) trained on synthetic images is used to assess visual realism by detecting drilling areas in real eggshell images. Finally, the accuracy of the DT is evaluated by experiments on a real eggshell.

**Results:**

The DAG model outperformed pitch modulation methods, achieving lower Frechet audio distance (FAD) and Frechet inception distance (FID) scores, demonstrating a closer resemblance to real drilling sounds. The CNN trained on synthetic images achieved a mean average precision (mAP) of 70.2 when tested on real drilling images. The DT had an alignment error of 0.22 ± 0.03 mm.

**Conclusion:**

A multimodal DT has been developed to simulate the creation of the cranial window on an eggshell model and its realism has been evaluated. The results indicate a high degree of realism in both the synthetic audio and images and submillimeter accuracy.

**Supplementary Information:**

The online version contains supplementary material available at 10.1007/s11548-025-03465-3.

## Introduction

Micro-drilling in neurosurgery enables access to deep brain areas while minimizing tissue damage. For example, in neuroma removal by drilling the temporal bone near the inner ear [1] and in pituitary tumor resection by accessing the pituitary gland through the sphenoid bone [2]. Precision is essential to navigate nerves and blood vessels safely, as errors can cause serious complications. Controlling drilling force, angle, and heat is critical, highlighting the importance of mastering these techniques for successful surgical outcomes [3].

Micro-drilling is also a crucial component of the cranial window technique used in neuroscience research on mice. This procedure involves creating a precise skull opening to allow direct access to the brain for visualization and manipulation in vivo [4], such as organ transplantation of the human brain [5]. This technique presents significant challenges, including variations in the shape and thickness of the mouse skull and the risk of heat damage to delicate brain structures [6], which require extensive training to ensure safe and reliable execution by researchers.

Several robotic systems have been proposed to assist cranial window creation in mice, offering precision beyond human capability, improved reproducibility, and higher experimental quality. Pak et al. [7] developed a robotic system that halts drilling when impedance changes are detected, preventing brain tissue damage after skull penetration. Ghanbari et al. [8] introduced Craniobot, a cranial microsurgery platform combining automated skull profiling with CNC milling for various procedures. Marinho et al. [9] introduced a robotic system featuring a manipulator equipped with a micro-drill for teleoperated cranial window creation.

Despite advances in robotic systems, these still rely on human input for precision and safety. Significant efforts are focused on developing fully autonomous systems for cranial window creation. AI-based methods are promising for enabling autonomous robotic drilling. Zhao et al. [10] proposed an AI-based drilling completion estimator and robot controller evaluated on an eggshell model considering the similar properties of eggshells to mouse skulls [11]. However, training these AI models requires extensive labeled data whose preparation is time-consuming.

To address these challenges, virtual reality (VR) simulators and digital twins (DT) have been proposed. While VR simulators provide immersive training environments without real-time data integration, A DT offers real-time virtual models of physical objects, continuously synchronized with their real-world counterparts for monitoring and optimization, enabling highly realistic simulations and synthetic data generation. This approach allows researchers to create robust datasets for training and testing autonomous robotic systems, reducing the need for live animals, minimizing ethical concerns, lowering costs, and facilitating impractical or impossible scenarios.

Research on cranial window simulation remains limited, but various VR and DT simulators for bone drilling have been documented. Shu et al. [12] developed a DT system for skull base surgery that combines optical tracking with real-time drilling simulation, enabling mixed-reality guidance. Several VR simulators for temporal bone dissection [13–15] have been designed to enhance surgical training, incorporating force and auditory feedback. Munawar et al. [16] introduced an immersive VR simulator for skull base surgery, offering real-time drilling simulation, haptic and auditory feedback, and ground truth data generation. Marinho et al. [9] created a DT platform for robotic AI exploration, featuring simplified drilling simulations on eggshells and mouse skulls, though it lacks volumetric drilling and auditory feedback.

Drill sounds provide real-time feedback during drilling, with changes in pitch, tone, or volume indicating proximity to critical boundaries or layer transitions. Robotic systems use microphones or acoustic sensors to capture these sounds, enabling dynamic adjustments to parameters like speed, depth, and force. AI models, such as the radial basis function (RBF) network employed by Ying et al. [17], have effectively used auditory feedback for robotic bone cutting, achieving around 95% accuracy.

VR simulators for bone drilling often use pitch modulation for auditory feedback through frequency synthesis [13, 14], spectral modeling [18], or audio playback [15, 19]. However, this method faces limitations, including modeling inaccuracies, scalability issues, and time-consuming adjustments. Additionally, it often fails to capture essential frequency components, making it unsuitable for generating realistic synthetic audio for training AI in autonomous drilling.

This work addresses the limitations of existing VR and DT simulators for micro-drilling, particularly in cranial window creation. We present a novel DT with realistic rendering and auditory feedback replicating micro-drilling on an eggshell model. The contributions include developing advanced synthetic imaging and audio and evaluating its effectiveness in replicating realistic visual and auditory cues essential for training autonomous robotic drilling in a simulated environment.

## Materials and methods

**Fig. 1:**
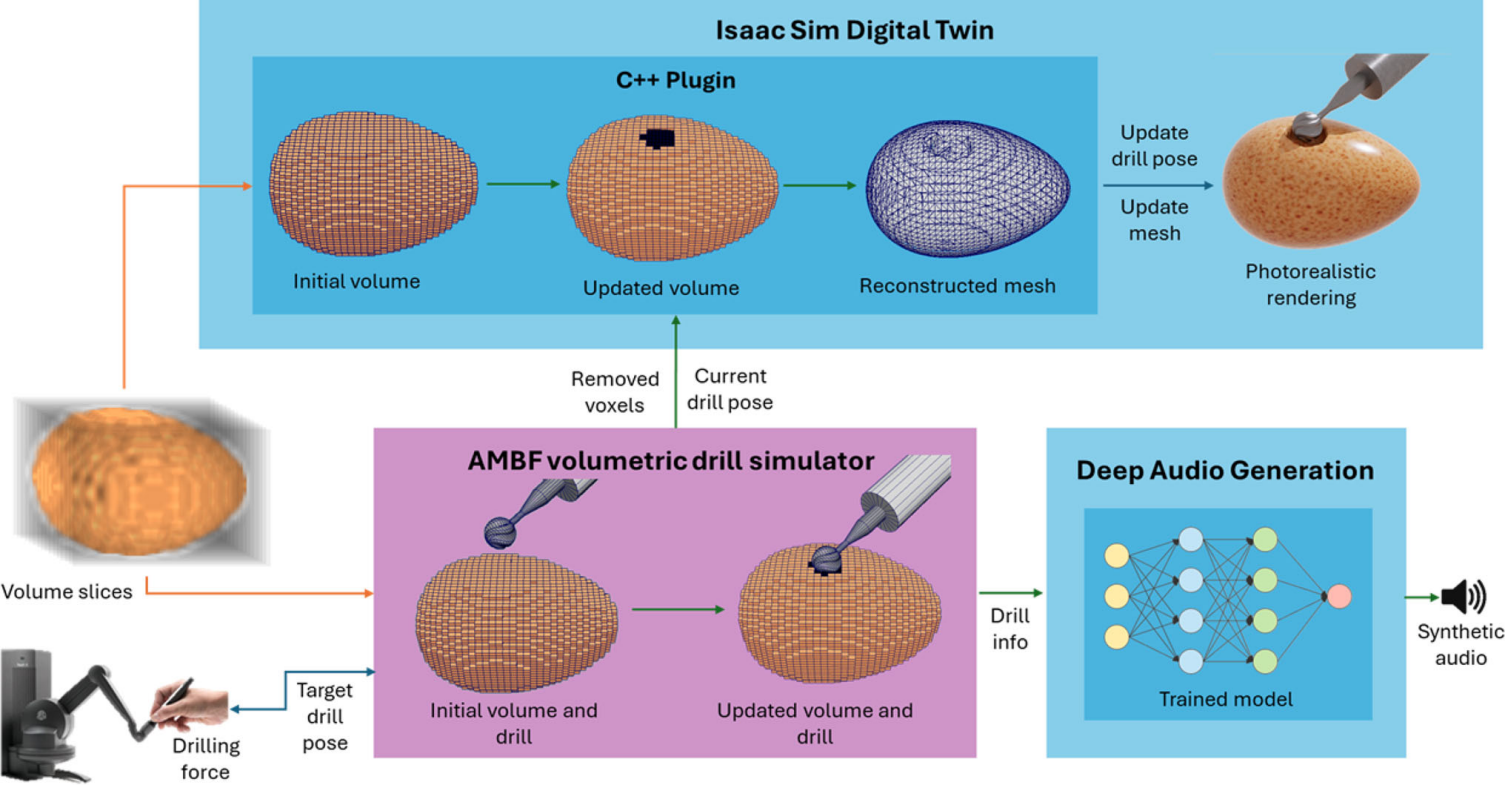
Overview of the system. The resolution of the eggshell model was lowered, and the drill size was enlarged for illustrative purposes

An overview of the proposed multimodal DT exemplified for an eggshell model is shown in Fig. 1. A simulator implements real-time volumetric drilling with haptic feedback. Data from this simulator are fed into the DT, updating the visual model and generating photorealistic images. An additional module synthesizes drilling sounds using a generative model. Each subsystem is detailed in the following subsections.

### Digital twin

**Fig. 2:**
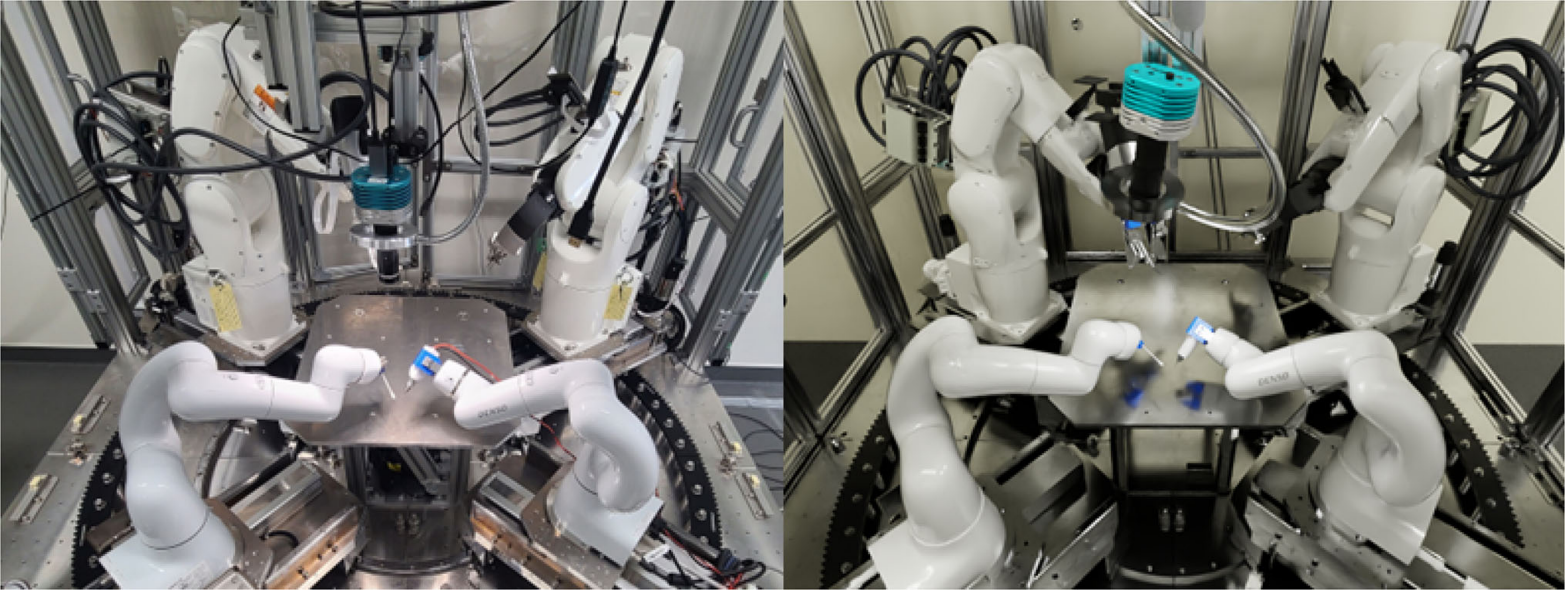
(Left) Robotic platform and its (right) digital twin

This work builds on the DT developed by Marinho et al. [9] using NVIDIA’s Isaac Sim [20], a robotics simulation platform that combines real-time ray tracing and physics-based simulations. Supporting complex virtual environments through the Universal Scene Description (USD) format, Isaac Sim enables detailed specifications of geometry, materials, lighting, and other properties for realistic simulations. The DT replicates a robotic system designed for autonomous scientific tasks, featuring four robotic arms on a circular rail with customized end effectors for precise, millimeter-level manipulation (see Fig. 2 left). It has been tested on tasks like teleoperated cranial window creation in ex vivo mice [9] and autonomous drilling in eggshell models [10].

The DT employs the exact CAD models of the physical robotic platform to ensure an accurate virtual representation. Each robotic arm is modeled as rigid bodies connected by actuated kinematic joints, replicating the structure, joint limits, and functionality of its physical counterpart. Visual materials and lighting were fine-tuned to match the appearance of the physical system, resulting in a highly detailed simulation that closely mirrors the real-world robotic platform (see Fig. 2 right).

### Volumetric drilling simulator

The asynchronous multi-body framework (AMBF), developed by Munawar et al. [19], is used for real-time volumetric drilling simulation in a voxel-based representation. Material removal is simulated by eliminating voxels contacted by a virtual 2 mm diameter surgical micro-drill burr controlled via a Touch X haptic device (3D Systems, USA) with a 1/3 motion scaling factor. The AMBF simulator provides contact data, including forces and voxel indices removed during drilling, with force feedback rendered to the operator through the haptic device. This feedback incorporates Gaussian noise to mimic drill motor vibrations, scaled to match empirical force measurements from eggshell drilling tests.

**Fig. 3:**
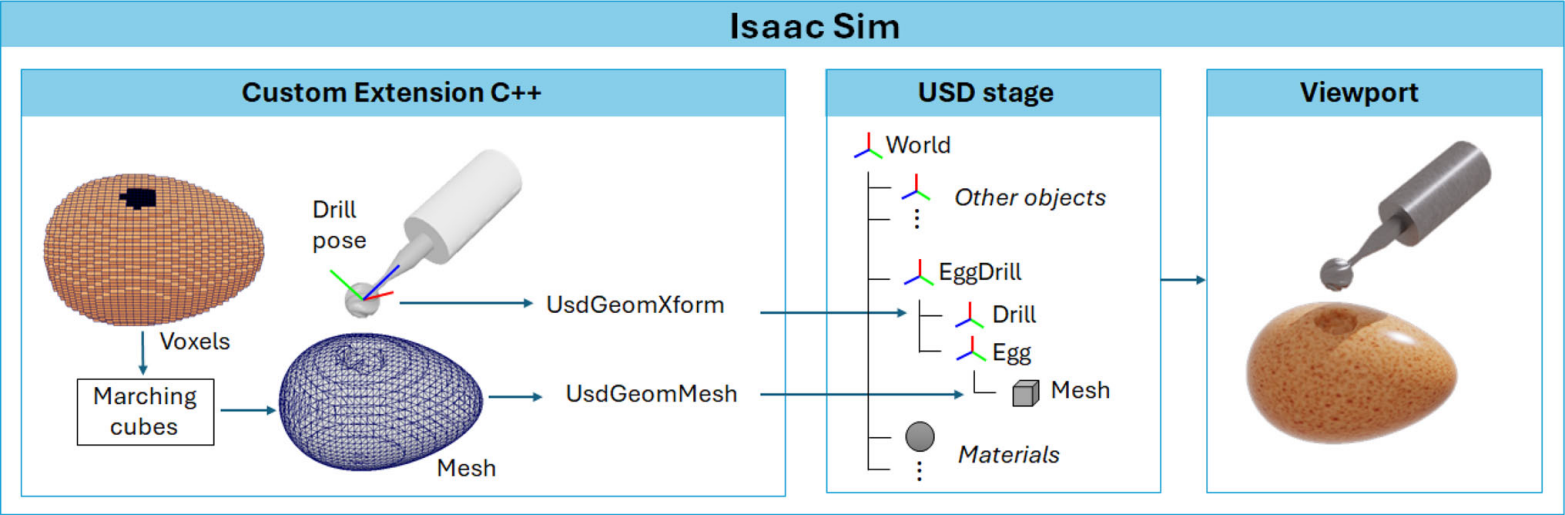
Overview of the Isaac Sim custom extension and USD stage update

The AMBF simulation and the DT in Isaac Sim are synchronized by updating the volume model and drill pose in real time. Removed voxel indices and drill pose are transmitted via UDP to a custom C++ plugin in Isaac Sim. The virtual robotic arm holding the drill is dynamically adjusted using a high-stiffness 6-DOF kinematic joint to align with the drill pose in the AMBF simulator. The volume model inside the plugin is updated with the removed voxel indices and the marching cubes algorithm [21] is employed to generate a isosurface triangle mesh within the USD stage for visualization. This workflow inside Isaac Sim is depicted in Fig. 3.

**Fig. 4:**
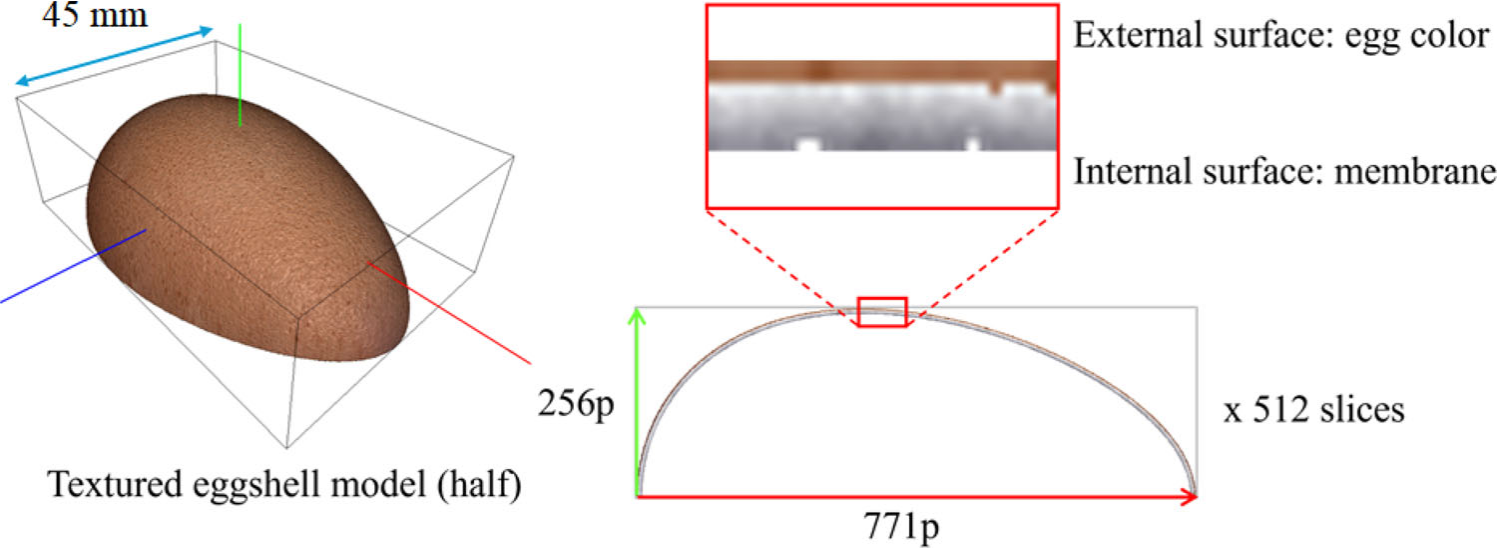
Eggshell model and detail of the colored voxels

The simulated eggshell was built following a parametric model of the shape of a chicken egg [22]. For efficiency, only the upper half visible to the operator from a top-view camera is considered. It is discretized into a voxel structure with 512 slices at a resolution of 271 $\times$ 256 pixels. Each slice is encoded into RGBA images, where the RGB channels indicate voxel color and the alpha channel represents density. The shell has an average thickness of 8 voxels, with the outer two layers textured with a real egg image for realism. Internal voxels display a grayscale gradient to simulate the color change during drilling (See Fig. 4).

### Deep audio generator

**Fig. 5:**
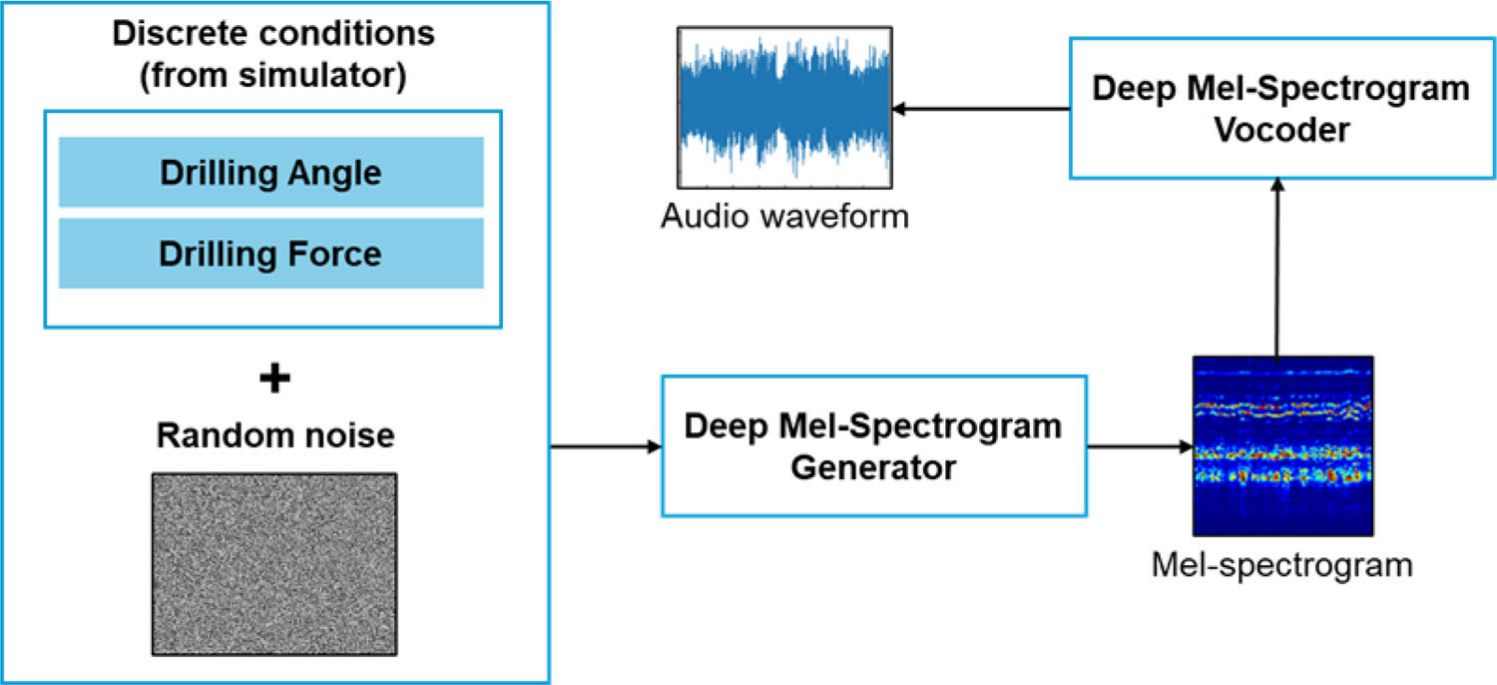
DAG pipeline

The proposed drilling audio generator, called the deep audio generator (DAG), is a two-stage generation pipeline (see Fig. 5) featuring a generator and a vocoder (inverter).

The generator model is inspired by CGAN [23] and DCGAN [24], and generates a magnitude mel-spectrogram given a latent vector and discrete drilling conditions. The key differences between the proposed model and DCGAN are: (1) One-hot encoded conditions are embedded and concatenated to the inputs of the generator and discriminator. (2) In the generator, batch normalization is applied to all except the last transpose convolution layers. In the discriminator, all convolution layers have instance normalization. (3) All ReLU activation functions are replaced with LeakyReLU. (4) There is no activation function at the output layer of the discriminator and the generator.

**Fig. 6:**
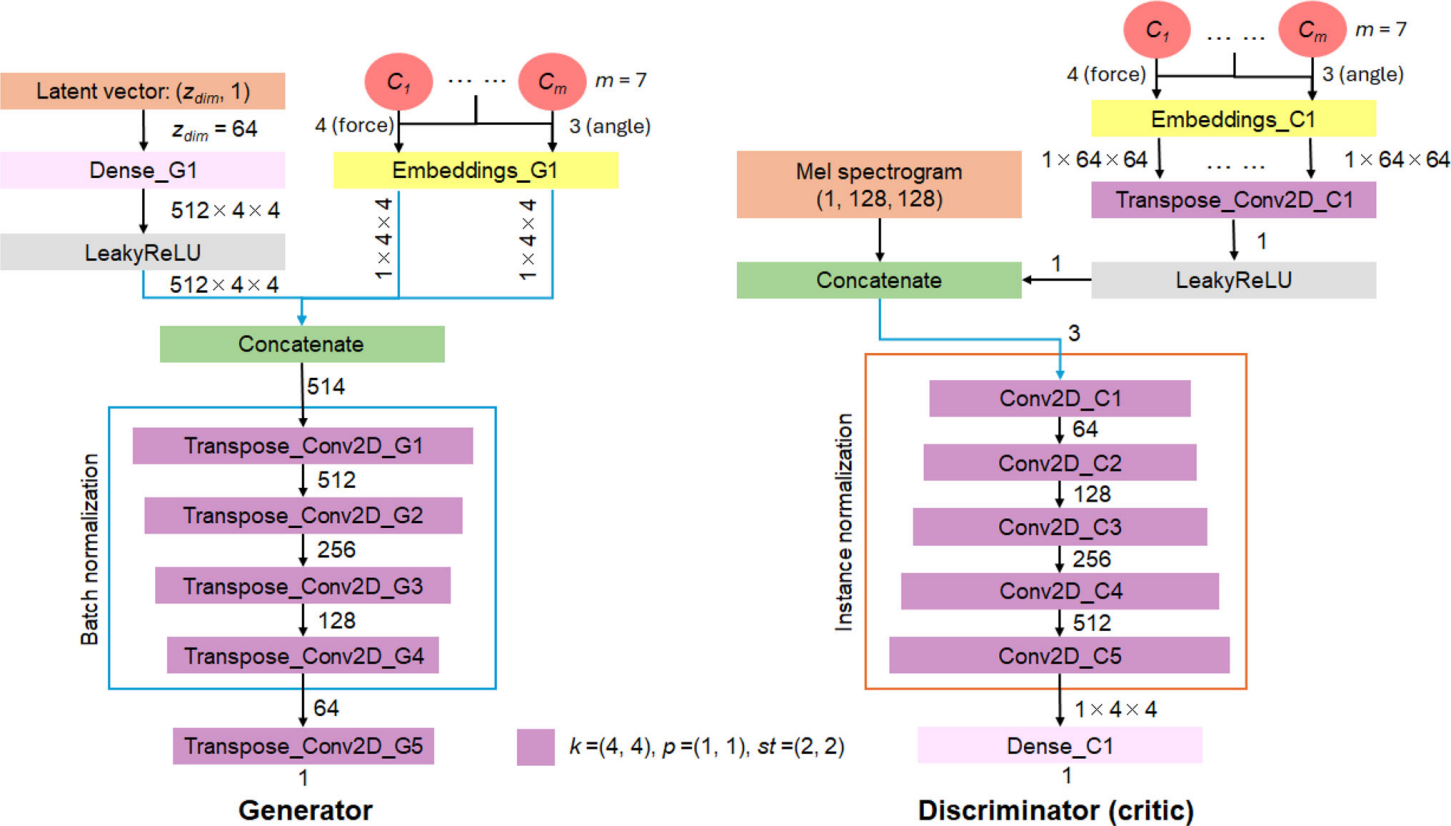
Full architecture of CWGAN-GP. Each LeakyReLU has a leakage coefficient of 0.2. The kernel size, padding, and stride are represented as *k*, *p*, and *st*, respectively

HiFi-GAN [25] is used as the vocoder to convert generated mel-spectrograms into audible waveforms, offering high fidelity and real-time inference. Unlike other audio processing methods, spectral normalization was not applied due to training convergence issues. To prevent mode collapse in the generator, the Wasserstein loss with gradient penalty ins employed instead of the traditional min–max strategy, referred to as CWGAN-GP generator in this work, with the discriminator functioning as a critic (see Fig. 6).

#### Data acquisition, preprocessing, and augmentation

The CWGAN-GP is trained with mel-spectrograms segregated based on the drilling angle and the drilling force. To build the dataset force and audio were collected on eggshell drilling experiments. Four drilling force classes (*n* none, *w* weak; *m* medium, and *s* strong) and three drilling angle classes (*V* vertical $\sim 90^{\circ }$, *L* large $45^{\circ }<\theta <90^{\circ }$, and *S* small $<45^{\circ }$) are defined. The drilling force class solely depends on the vertical force as it is the most dominant force component, consistent with that reported in [26]. The collected audio recordings are sliced into clips of 2 s and converted into 128 x 128 mel-spectrograms without empty gaps compatible with CNN operations using the TorchAudio library. The dataset is augmented by recursively applying time transposition where two segments of the audio are swapped at a randomly selected point.

#### Model training

The CWGAN-GP is trained with mel-spectrograms, whereas the HiFi-GAN is trained with both mel-spectrograms and audio waveforms. The CWGAN-GP, a Wasserstein GAN, requires updating the critic more frequently than the generator, with $N_{critic}=5$ number of critic updates per batch. The gradient penalty $\lambda =10$ constrains the generator-critic loss function for gradient stability. As for the HiFi-GAN, the “Config V2” parameters as prescribed in the original paper [25] are used. The validation step is only performed for HiFi-GAN by comparing the mel-spectrograms of the real and generated audio waveforms. The models were trained using an Adam optimizer (Learning rate: 0.00005, Beta1: 0.5, Beta2: 0.9) for 1000 and 3100 epocs respectively. For HiFi-GAN, a learning rate decay of 0.999 was set and 20% of the dataset was employed for validation. For CWGAN-GP, the model at epoch 1000 was used. As HiFi-GAN performs best between 110k and 135k iterations, with degradation after 200k iterations, the model at 135k was chosen.

### Synthetic images for training CNN

Synthetic images and corresponding masks generated by the volumetric drilling simulator are used to train a CNN model, allowing the verification of its visual validity. The underlying concept is that if a model trained on synthetic images and masks can accurately predict results on real images, the visual performance of the volumetric drilling simulator can be considered comparable to that of real images. Drilling area detection on the eggshell model, identified as a significant subtask of autonomous robotic drilling in [10], is chosen as the testing task. The following sections discuss the creation of synthetic images, the preparation of training and testing datasets, the network architecture, and the model training process.

#### Synthetic image creation

**Fig. 7:**
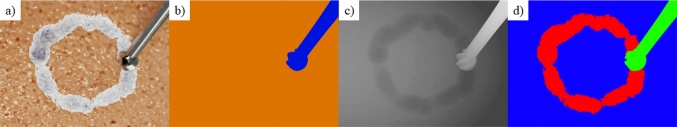
Synthetic images generated using Isaac Sim for a single simulation frame: **a** color image, **b** semantic segmentation label, and **c** depth map. **d** Segmentation masks for the drill (green), drilled (red), and undrilled (blue) zones

Isaac Sim can generate synthetic color images, semantic segmentation labels, and depth maps (see Fig. 7a,b,c). However, it cannot provide per pixel semantic labeling of drilled and undrilled areas because the volumetric model is visualized as a single triangle mesh of the isosurface, assigned a uniform semantic label. To overcome this limitation, we developed a post-processing method that combines segmentation labels with depth images. By comparing the initial and current depth maps, pixels that are not part of the drill and have increased depth are considered drilled zones (see Fig. 7d).

**Table 1:** Details of hardware requirements

	Volumetric drilling simulator	Digital twin	Deep audio generator	CNN for drill area detection
Employed				
CPU	Core i9	Core i9	Core i9	Core i9
GPU	RTX TITAN	RTX 6000 ($\times$2)	RTX TITAN	RTX A5000
Minimum requirements				
CPU	Core i5	Core i7	Core i5	Core i5
GPU	RTX 3060	RTX 3070	RTX 3060	RTX 3060
Can run on edge devices				
	N/A	N/A	Yes	Yes
Cost*	Low	High	Low	Low
Benefit	Interactive drilling simulation	Photorealistic rendering	Realistic audio generation	For autonomous robotic drilling

*Relative to the estimated cost of a basic workstation for machine learning research (2,500–5,000 USD as of 2025)

#### Dataset preparation

A dataset of 6,286 synthetic color images and their segmentation masks was collected from a volumetric drilling simulator. Ground truth bounding boxes, represented as 4D vectors ($x_{1}$, $y_{1}$, $x_{2}$, $y_{2}$), were derived from segmentation masks by identifying the minimum and maximum pixel values in the drilled area, with a 10-pixel offset for accuracy. To improve robustness against variations in brightness and contrast due to real-world conditions, image augmentation was applied, doubling the dataset to 12,572 images. The dataset was split: 80% for training, 10% for validation, and 10% for testing, ensuring the performance of the model before testing on real images. Additionally, 518 realistic images from eggshell drilling experiments with manually labeled bounding boxes were collected for final testing.

#### Network architecture

A CNN consistent with the previous work [10] is used, featuring ResNet-50 [27] as the backbone, with convolutional, pooling, and deconvolutional layers. The network has two branches: drilling area detection and completion level recognition. For this task, only the detection branch is trained, enhanced with residual structures and multi-scale feature maps for classification and regression. A conv-skip connection processes feature layer outputs to generate the drilling area bounding box.

#### Model training

The loss function is the same as [28], which is a weighted sum of the localization loss and the confidence loss. During the training stage, the images are trained in a total of 6,000 epochs with the Adam optimizer. The training rate is set as $0.01\times 0.9995^{n}$ while *n* is the number of epochs. Moreover, we used a batch size of 16 during the training stage. All the architectures were implemented using PyTorch.

The details of the hardware used for each component and the minimum hardware requirements are summarized in Table 1.

### Real egg drilling experiment

**Fig. 8:**
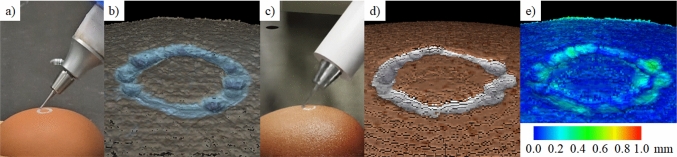
**a** Drilling on a real eggshell and **b** captured point cloud after drilling. **c** Replicated drilling in DT and **d** simulation point cloud. **e** Point cloud alignment error

**Fig. 9:**
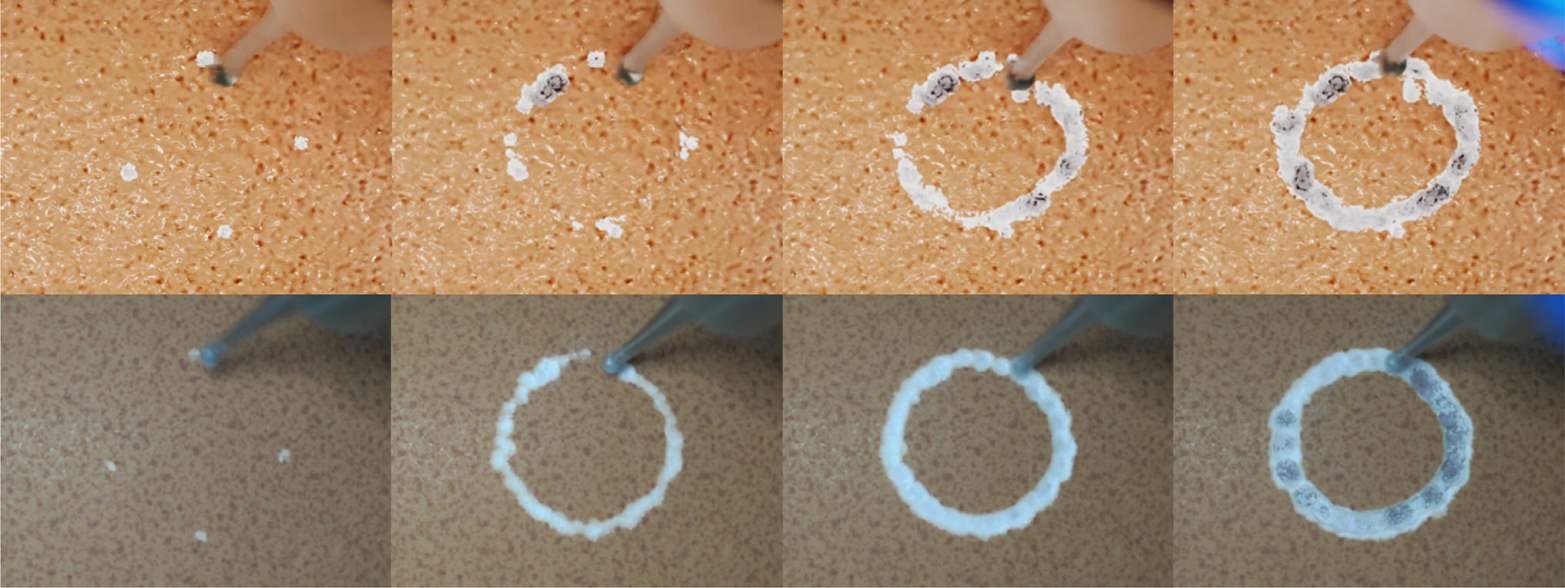
Screenshot of eggshell drilling simulation in the DT (top), and the corresponding images from the experiment on a real egg (bottom)

The accuracy of the DT is evaluated by comparing the updated volumetric model in the simulator with a real eggshell after drilling using the real robot platform. This experiment is evidenced in Fig. 8. First, a chicken eggshell is drilled via teleoperation of the robotic drill, and the trajectory of the end effector, defined at the drill burr, is recorded. At the end of the operation, the point cloud of the drilled egg is obtained using a microscopic stereo camera system by Lin et al. [29] with an accuracy of 0.10 ± 0.02 mm. The recorded trajectory is then replayed in the DT. The position egg in simulation is manually adjusted to ensure that the initial four contacts made by the drill touch the surface of the eggshell (see Fig. 9).

## Results

The DT implemented using the Isaac Sim simulator for the generation of photorealistic images using real-time render can run at $\sim$80 FPS. Rendered frames for interactive drilling on the eggshell model and its real counterpart are shown in Fig. 9. A video provided as supplementary material shows interactive eggshell drilling in the DT demonstrating synthetic drill sounds for different force levels, and evidences the DT replicating the real egg drilling experiment. Quantitative evaluations of the realism of synthetic audio and images, as well as drilling accuracy in an eggshell scenario, are presented in the following subsections. The dataset used for training and evaluation is publicly available in an online repository.^1^

### Deep audio generator

**Fig. 10:**
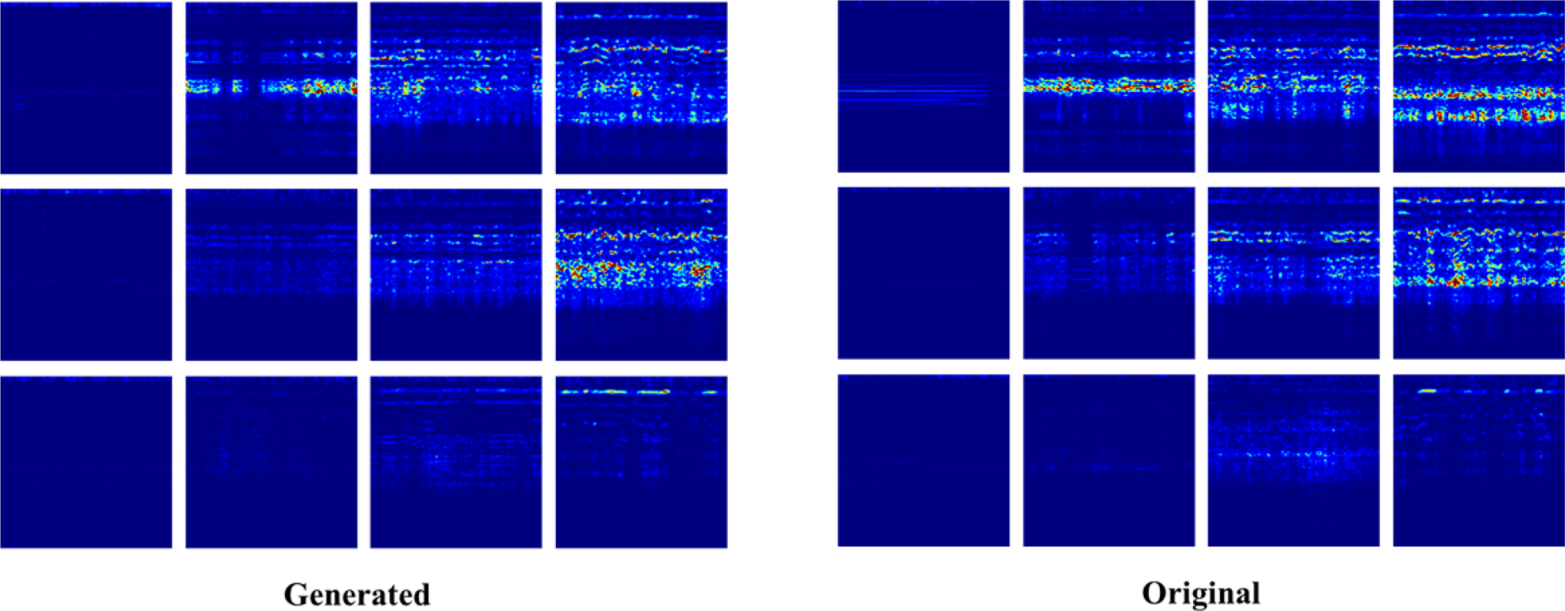
Mel-spectrogram samples of DAG (left) and the original (right). For each group, the column progresses to the right in increasing drill force (*n* to *s*), while the row progresses downwards with increasing drill angle (*S* to *V*)

Qualitative visualizations show that the mel-spectrograms of the waveforms generated by DAG closely resemble their original counterparts (see Fig. 10). Minor variations in mel-spectrogram patterns are expected due to the inherent noisiness of drill sounds, which is important for generating non-deterministic audio. However, significant deviations that alter the overall pattern and produce audio indistinguishable from the intended class are undesirable. The results demonstrate that the generated mel-spectrograms do not exhibit such unwanted macro-variations.

Following the pitch modulation implementation by Munawar et al. [19], the Frechet audio distance (FAD) [30] and Frechet inception distance (FID) [31] scores are calculated for both methods. FAD and FID scores compare the distribution of feature embeddings between real and generated waveforms and mel-spectrogram sets, respectively. FID and FAD scores, computed on batches of 30 samples, are reported in Table 2, showing that the proposed method achieves lower scores, indicating closer similarity between the generated and real drilling sounds.

### Synthetic images for training CNN

**Fig. 11:**
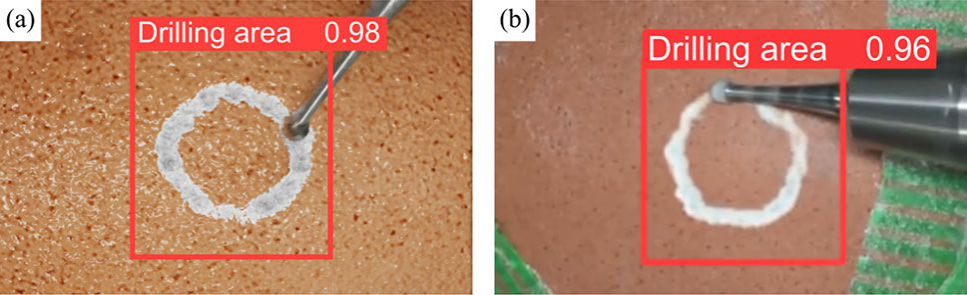
Samples of network testing result trained on synthetic images. **a** Tested on a synthetic image (not included in the training dataset); **b** tested on a real image

Mean average precision (mAP) is applied as the evaluation metric of the drilling area detection task on a dataset of 1,658 synthetic and real images. The testing result on synthetic images (not included in the training dataset) can reach up 81.3 in mAP while 70.2 in mAP for testing on real images (see Fig. 11). Considering the errors when manually marking the coordinates of the bounding box, the result is within acceptable limits. Moreover, both the testing speeds on synthetic images and real images can reach 77 Hz, enabling real-time detection. Taken together, the results validate the simulator’s veracity in visual terms.

### Real egg drilling experiment

After completing the drilling in the DT, the point cloud of the voxel surface was obtained. To assess accuracy, the point clouds were aligned using the iterative closest point method, and the Chamfer distance was obtained. The alignment error for ten trials was 0.22 ± 0.03 mm, and an example of point cloud alignment is shown in Fig. 8e.

## Discussion

The DT simulates drilling on volumetric models of an eggshell, mimicking the bone drilling required for cranial window creation in mice. The voxel-based simulation, with a resolution of 0.088 mm per voxel (512 slices), provides sufficient detail for a 2 mm drill burr and an 8 mm cranial window. However, the volumetric drilling simulation model has certain limitations. The voxels behave as a single rigid body, which prevents the representation of bone flap removal upon completion of drilling. Additionally, it incorporates a uniform stiffness property solely for force feedback computation, without accounting for soft body deformations.

**Table 2:** FID and FAD scores for pitch modulation and DAG. The drill angle class is fixed at class *S*

Drill force	Pitch modulation		DAG	
	FID	FAD	FID	FAD
Weak	74.94	5.86	40.13	4.45
Medium	91.53	52.88	35.19	11.75
Strong	92.69	162.74	41.49	35.17

For audio realism, the proposed DAG model generates high-fidelity waveforms with low FID and FAD scores, making real and synthetic drilling audio indistinguishable. Waveforms are generated in 6.4 ms on a GPU and 44 ms on a CPU, ensuring seamless audio transitions at 60Hz. A limitation is that the model can only synthesize sounds based on its training data. Since real cranial window audio from mice is unavailable, the model generates sounds based on eggshell drilling but can be retrained with appropriate samples. In this study, 12 drilling conditions, combining four forces and three angles, were sufficient to produce realistic sounds, though additional classes would require more extensive data collection.

Using Isaac Sim’s ray tracing, photorealistic images were generated, accurately reflecting drilling on an eggshell, including color changes as voxels were removed (see Fig. 9). A CNN trained on synthetic images successfully detected real drilling areas via data augmentation. Nevertheless, these results cannot be directly generalized to mouse skulls due to differences in shape, color, and unmodeled factors such as bone dust formation and bleeding. Although the simulation model accurately replicates drilling in the eggshell model, it does not realistically represent the layered tissue structure of the real mouse cranium, which also includes flexible bone tissue. Future work will address these limitations by incorporating improved physics engines and real mouse data.

The experimentally measured accuracy of the DT was 0.22 ± 0.03 mm. Although this represents submillimeter accuracy, the relative error is significant compared to the drill burr size (2 mm) and the eggshell thickness (0.33-$-$0.36 mm). This inaccuracy likely arises from errors in the drill trajectory measured using robot kinematics, including compliant joints, backlash in the end effector and tool attachment, and other assembly inaccuracies. During teleoperation, the operator closes the control loop using visual cues to mitigate these errors. However, at present, we lack a method to obtain precise drill trajectories. To address this limitation, future work will incorporate a microscope stereo camera system to track the actual drill position.

Regarding the hardware requirements of the digital twin, Table 1 provides both the specifications of the hardware used in this work and the minimum required specifications. The most prohibitive component is the DT based on Isaac Sim, which requires a high-end GPU to achieve photorealistic rendering. However, if photorealism is not essential, interactive drilling can be performed at a lower cost using basic visualization in the AMBF simulator. While the cost of the haptic device is not discussed here, it can be omitted when drilling playback data is available. For audio generation and drilling area detection, although a high-spec GPU can accelerate the training process, the deployment of the trained model can be executed on edge devices allowing flexible and cost-effective application.

## Conclusion

In this work, a multimodal DT simulator was developed to replicate simulated cranial window creation on an eggshell model. The simulator generates highly realistic visual data and synthetic audio, offering a comprehensive simulation environment. The enhanced realism of the DAG model was demonstrated by comparing it with traditional pitch modulation methods, showing superior fidelity in reproducing drilling sounds. The realism of the synthetic visual data was further validated by training a CNN to autonomously detect drilling areas for autonomous robotic drilling. The model trained entirely on synthetic images was successfully applied to real-world images of eggshell drilling.

This DT represents a significant step toward generating multimodal synthetic data that is crucial for training AI models, which are essential to advance robotic autonomy in complex surgical tasks. By simulating both visual and auditory feedback, this platform can provide a rich dataset for developing and fine-tuning AI algorithms, paving the way for more sophisticated and autonomous robotic systems in the future.

## Supplementary Information

Below is the link to the electronic supplementary material.

Supplementary file 1 (mp4 67085 KB)

## Footnotes

1 Refer to https://github.com/SaulHP91/digital_twin_egg_data for more information.
